# Relationships between plant species richness and grazing intensity in a semiarid ecosystem

**DOI:** 10.1002/ece3.10668

**Published:** 2023-10-31

**Authors:** Timothy E. Fulbright, J. Alfonso Ortega‐Santos, Stacy L. Hines, Dillan J. Drabek, Ramon Saenz, Tyler A. Campbell, David G. Hewitt, David B. Wester

**Affiliations:** ^1^ Caesar Kleberg Wildlife Research Institute MSC 218, Texas A&M University‐Kingsville Kingsville Texas USA; ^2^ Texas A&M AgriLife Extension, Department of Rangeland Wildlife and Fisheries Management Corpus Christi Texas USA; ^3^ Natural Resources Conservation Service Claude Texas USA; ^4^ East Foundation San Antonio Texas USA

**Keywords:** intermediate disturbance hypothesis, Milchunas‐Sala‐Lauenroth model, spatial heterogeneity, South Texas, ungulates, vegetation

## Abstract

Plant species richness is an important property of ecosystems that is altered by grazing. In a semiarid environment, we tested the hypotheses that (1) small‐scale herbaceous plant species richness declines linearly with increasing grazing intensity by large ungulates, (2) precipitation and percent sand interact with grazing intensity, and (3) response of herbaceous plant species richness to increasing intensity of ungulate grazing varies with patch productivity. During January–March 2012, we randomly allocated 50, 1.5‐m × 1.5‐m grazing exclosures within each of six 2500 ha study sites across South Texas, USA. We counted the number of herbaceous plant species and harvested vegetation in 0.25‐m^2^ plots within exclosures (ungrazed control plots) and in the grazed area outside the exclosures (grazed treatment plots) during October–November 2012–2019. We estimated percent use (grazing intensity) based on the difference in herbaceous plant standing crop between control plots and treatment plots. We selected the negative binomial regression model that best explained the relationship between grazing intensity and herbaceous plant species richness using the Schwarz‐Bayesian information criterion. After accounting for the positive effect of precipitation and percent sand on herbaceous plant species richness, species richness/0.25 m^2^ increased slightly from 0% to 30% grazing intensity and then declined with increasing grazing intensity. Linear and quadratic responses of herbaceous plant species richness to increasing grazing intensity were greater for the least productive patches (<15.7 g/0.25 m^2^) than for productive patches (≥15.7 g/0.25 m^2^). Our results followed the pattern predicted by the intermediate disturbance hypothesis model for the effect of grazing intensity on small‐scale herbaceous plant species richness.

## Graphical abstract

We examined the relationship between intensity of grazing by large ungulates and herbaceous plant species richness in a semiarid environment. Species richness increased with increasing grazing intensity up to around 30% utilization of herbaceous vegetation. Response to grazing followed a hump‐backed relationship with herbaceous plant species richness declining with increasing intensity of grazing exceeding 30% utilization.

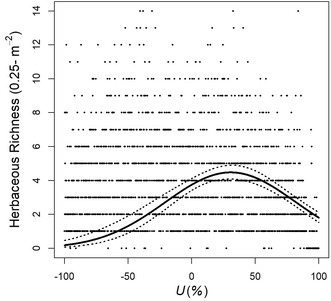

## 1 INTRODUCTION

Plant species richness and diversity are important for stable functioning of ecosystems, productivity, and resistance to invasive species (Brown et al., 2007; Knops et al., 2002). In grasslands, plant species richness often depends on the intensity of grazing by large native or domestic ungulates (Baaker et al., 2006; Herrero‐Jáuregui & Oesterheld, 2018; Pykälä, 2004). The intermediate disturbance hypothesis provides the basis for a model (IDH model) widely proposed in the literature regarding the nature of the relationship between plant species richness and grazing intensity (Fedrigo et al., 2017; Gao & Carmel, 2020a, 2020b; Li et al., 2021; Sasaki et al., 2009; Veblen et al., 2019). Briefly, the intermediate disturbance hypothesis proposes that intermediate levels of disturbance reduce the competitive ability of dominant species, allowing less competitive species to occupy a site, thereby increasing species diversity (Connell, 1978; Li et al., 2021; Oba et al., 2001).

According to the IDH model, plant species richness increases with increasing grazing intensity up to some moderate intensity of grazing and then declines thereafter, yielding a hump‐shaped quadratic response curve. Veracity of the intermediate disturbance hypothesis is equivocal (Fox, 2013). One of the criticisms of the IDH model is that mechanisms other than competition may be involved in vegetation responses to grazing such as increased tillering, seed transport by animals, nutrient redistribution from excreta, and compensatory growth of grazed plants (Akhazari et al., 2015; Milchunas et al., 1988; Oba et al., 2001).

Habitat productivity, climate, soil properties, and spatial scale may influence the relationship between species richness and grazing intensity (Baaker et al., 2006; Gao & Carmel, 2020a, 2020b; Lezama et al., 2014; Milchunas et al., 1988; Olff & Ritchie, 1998; Whittaker et al., 2001). Moderate intensities of grazing may result in greater species richness in subhumid grasslands but not in dry environments (Milchunas et al., 1988). In dry environments, species richness declines linearly along a continuum from no grazing to intensive grazing because plants mainly compete for soil resources instead of light resources as occurs in productive subhumid grasslands. A decline in species richness with increasing grazing intensity in dry areas follows the Milchunas‐Sala‐Lauenroth (MSL) model (Gao & Carmel, 2020a, 2020b; Milchunas et al., 1988). The decline in species richness may be slow because of compensatory growth of vegetation (Oba et al., 2001). A more rapid decline in species richness is expected to occur with increasing grazing intensity in areas with a short evolutionary history of herbivory (Milchunas et al., 1988; Oba et al., 2001).

Variable findings reported in the literature make it unclear whether responses of plant species richness to grazing fit general patterns predicted by the IDH or MSL models (Lezama et al., 2014). In a review of 63 studies, the IDH model prediction of a hump‐shaped curve was supported in wet areas but in dry areas species richness declined with increasing grazing intensity (Gao & Carmel, 2020b). Evolutionary history of grazing was unrelated to plant responses. Another meta‐analysis of 48 studies found that the effects of increasing grazing intensity on plant species richness ranged from −22% to +28% (Herrero‐Jáuregui & Oesterheld, 2018). The most negative responses to increasing grazing intensity occurred in arid systems with low plant productivity.

Plant species richness is often related to soil texture (Pennington et al., 2017; Sanaei et al., 2019). Percent sand and grazing intensity interacted to influence standing crop of forbs in a study in South Texas (Fulbright et al., 2021). Soil texture and grazing intensity may also interact to influence plant species richness but effects were not examined in the South Texas study.

Spatial heterogeneity in vegetation and in grazing by herbivores are often given little consideration in studies of grazing effects on plant species richness. Vegetation in semiarid environments typically consists of a mosaic of patches of denser vegetation interspersed with sparse patches or bare ground (Aguiar & Sala, 1999). Patches are discrete components of the landscape that differ from one another in vegetation structure and composition (Pickett & Cardenasso, 1995). Ungulates may forage in vegetation patches supporting greater biomass than surrounding vegetation patches (Hebblewhite et al., 2008; Li et al., 2021; Wilmshurst et al., 2000). For example, bison (*Bison bison athabascae*) avoided vegetation patches with biomass <120 g/m^2^ (30 g/0.25 m^2^ or 1200 kg/ha) and selected patches with 156–219 g/m^2^ (39–55 g/0.25 m^2^ or 1560–2190 kg/ha; Bergman et al., 2001). Effects of grazing on vegetation may be masked or less apparent when vegetation patches that ungulates avoid are included in analyses of grazing effects on plant biomass (Fulbright et al., 2021). Ungulate grazing intensity did not influence forb standing crop in South Texas when a data set that included all randomly sampled plots was included (Fulbright et al., 2021). The reason for the lack of influence of ungulate grazing was likely that ungulates avoided grazing in portions of the landscape with low productivity, when vegetation patches with grass standing crop ≥628 kg/ha (15.7 g/0.25 m^2^ or 62.8 g/m^2^) were analyzed forb standing crop declined linearly with increasing grazing intensity.

In our study, small‐scale plant species richness is diversity at the micro‐habitat level following terminology used by Brown et al. (2007). Habitat productivity was based on the standing crop of herbaceous vegetation, with patches supporting <15.7 g/0.25 m^2^ (50 g/m^2^ or 628 kg/ha) within grazing exclosures defined as unproductive patches that ungulates avoid based on results of Fulbright et al. (2021) and patches with ≥15.7 g/0.25 m^2^ defined as the most productive habitat patches on the landscape.

Our objective was to test the hypotheses that (1) small‐scale herbaceous plant species richness in the semiarid environment of our study area declines linearly with increasing grazing intensity by large ungulates, (2) precipitation and soil texture (percent sand) interact with grazing intensity, and (3) the effect of increasing ungulate grazing intensity on herbaceous plant species richness varies with patch productivity. We predicted that small‐scale herbaceous plant species richness is more strongly and negatively related to increasing ungulate grazing intensity in productive patches than in sparse patches of vegetation because ungulates appear to avoid grazing in patches with <15.7 g/0.25 m^2^ herbaceous standing crop (Fulbright et al., 2021). Including only patches that are most likely to be grazed should result in a stronger relationship with grazing intensity. Also based on the findings of Fulbright et al. (2021), we predicted that the effect of increasing grazing intensity on small‐scale herbaceous plant species richness would be stronger with higher percent sand and precipitation.

## 2 METHODS

### 2.1 Study area

**FIGURE 1:**
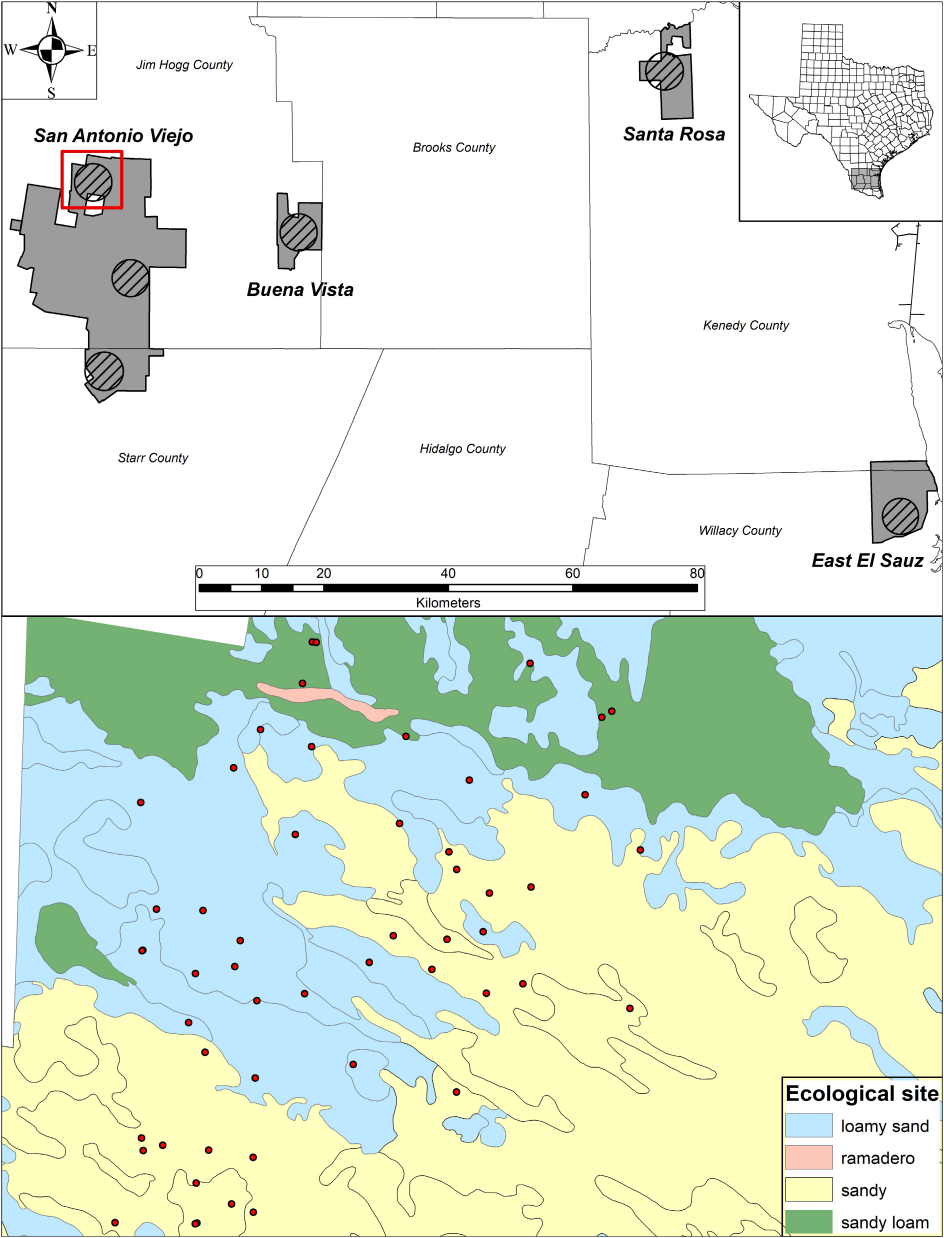
Top panel: Location of six 2500 ha study sites (cross‐hatched circles) on the San Antonio Viejo, Buena Vista, Santa Rosa, and East El Sauz ranches in South Texas, USA. Bottom panel: locations of paired exclosures and grazed plots (red dots) on the north site (inside red square) of the San Antonio Viejo ranch in 2012 illustrating the dispersion of paired plots among ecological sites (box on lower right of figure).

We conducted research on six 2500 ha study sites 10–134 km apart within four ranches operated by the East Foundation, an Agricultural Research Organization that promotes advancement of land stewardship through ranching, science, and education (Figure 1). Study sites were on the Buena Vista Ranch in Jim Hogg County, Texas, USA (6113 ha; lat 26°57′14.4″ N, long −98°27′21.6″ W); El Sauz Ranch in Willacy County, Texas, USA (10,984 ha; lat 26°31′58.8″ N, long −97°29′23.9″ W); and Santa Rosa Ranch in Kenedy County, Texas, USA (7544 ha; lat 27°10′58.8″ N, long −97°51′39.6″ W). Three study sites were on San Antonio Viejo Ranch in Jim Hogg and Starr Counties, Texas, USA (60,034 ha; lat 27°1′44.4″ N, long −98°47′13.2″ W; lat 26°53′49.2″ N, long −98°43′40.8″ W; and lat 26°45′25.2″ N, long −98°46′11.9″ W). All study sites, except one on the San Antonio Viejo Ranch, were in the Coastal Sand Plain ecoregion (Diamond & Fulbright, 1990; Forman et al., 2009; Fulbright et al., 1990). One site on the San Viejo Ranch was in the Tamaulipan Thornscrub ecoregion (Fulbright, 2001).

Vegetation of two study sites on the San Antonio Viejo Ranch and on the Buena Vista Ranch was dominated by mesquite (*Neltuma glandulosa*) with understory clusters of spiny hackberry (*Celtis ehrenbergiana*) and other shrubs and areas of open grassland. The site in the Tamaulipan Thornscrub was dominated by blackbrush acacia (*Vachellia rigidula*). The Santa Rosa Ranch was dominated by mesquite with small forests of live oak (*Quercus fusiformis*) while the El Sauz ranch supported open prairies with scattered live oaks and coastal saline plant communities. Dominant soil series at the study sites include the Nueces‐Sarita association, Delmita, Comitas, Galveston, Mustang, Palobia, Sauz, Yturria, Copita, McAllen, and Zapata (USDA‐NRCS, 2011a, 2011b). Percent sand for soils across the study sites ranged from 52 to 100 ($\bar{x}$ = 90).

Climate of the study area is semiarid (Norwine & Bingham, 1986). Bimodal peaks in rainfall in the study areas occur during May–June and September–October (Fulbright et al., 1990). Monthly rainfall during our study ranged from 0 to 292 mm averaged across ranches (Fulbright et al., 2021). Long‐term coefficient of variation in annual (1932–2002) precipitation ranges from around 20% along the coast to 32% near the Santa Rosa Ranch and 37% near the Buena Vista and San Antonio Viejo ranches (Parylak, 2010).

Cattle (*Bos* sp.), nilgai (*Boselaphus tragocamelus*), and white‐tailed deer (*Odocoileus virginianus*) were the primary ungulate grazers on the ranches. Wild pigs (*Sus scrofa* L.) and collared pecarries (*Pecari tajacu*) were also present. Nilgai and wild pigs were exotic wildlife species, whereas white‐tailed deer and collared peccaries are natives. From 2013 to 2019, cattle stocking rate (ha/animal unit) on each of the four East Foundation ranches ranged from 14.9 to 25.1 ($\bar{x}$ = 19.2) for Buena Vista, from 14.5 to 36.6 ($\bar{x}$ = 19.8) for El Sauz, from 10.0 to 15.3 ($\bar{x}$ = 13.0) for Santa Rosa, and from 22.2 to 35.7 ($\bar{x}$ = 25.8) for San Antonio Viejo. Detailed estimates of densities of cattle, nilgai, and white‐tailed deer are in Fulbright et al. (2021).

### 2.2 Vegetation sampling

We sampled vegetation in the study sites in autumn (October and November) 2012–2019. During January–March 2012, we randomly allocated 50, 1.5‐m × 1.5‐m grazing exclosures using ArcMap GIS (ArcGIS software v. 10, ESRI, Redlands, CA) software within each of the six 2500 ha study sites. Grazing exclosures consisted of 10 cm × 10 cm spacing, six‐gauge galvanized utility panels held in place by 4 t‐posts that were spread out within each 2500 ha study site. Large ungulates were excluded from grazing inside exclosures but had access to consume forages outside of exclosures. We did not exclude small herbivores such as lagomorphs. We placed grazing exclosures in areas dominated by herbaceous vegetation. At the time we placed an exclosure we also selected a paired, grazed plot around 10 m from each exclosure. We selected paired plots that had visually similar vegetation and bare ground as plots within the exclosures. We sampled vegetation within and outside grazing exclosures after they were in place for 8–12 months on each study site. We sampled a total of 300 pairs of plots within grazing exclosures and outside grazing exclosures/year across the six study (50 pairs/site × 6 sites) sites and a total of 2400 (50 pairs/site × 6 geographical separate sites × 8 years) pairs of plots within grazing exclosures and outside grazing exclosures across study sites and years of study. Most of the herbaceous forage production in the region occurs during April–June and September–October. Autumn is the only season when grasses and forbs are concurrently in their peak growing season in south Texas (Fulbright & Ortega‐S, 2013).

We harvested standing crop of herbaceous vegetation at ground level within a 0.5‐m × 0.5‐m sampling frame in the center of each grazing exclosure (ungrazed control plots) and in each paired grazed sampling area (grazed treatment plots). Concurrently with harvest of standing crop, we counted the number of species of herbaceous plants within each sampling frame. Herbaceous plant species encountered during sampling are listed in the supplementary materials in Fulbright et al. (2021). We dried standing crop samples at 45°C until they reached a constant mass, and then, we weighed them to the nearest 0.1 g. After we completed sampling, we moved grazing exclosures 10 m in a randomly assigned cardinal direction (previously sampled locations were avoided) and we then selected and marked a new paired grazed area.

### 2.3 Precipitation and soils

We used historical rainfall records from PRISM Climate Data (prism.oregonstate.edu) to determine monthly precipitation for pairs of grazing exclosures and grazed plots within each study site. We extracted values for pairs of grazing exclosures and grazed plots of percent sand within each site from National Resource Conservation Service data (USDA‐NRCS, 2011a, 2011b).

### 2.4 Statistical analyses

We used a true paired experimental design with each randomly allocated paired sampling location (i.e., grazed and corresponding ungrazed area) defined as the experimental unit (Hines et al., 2022). To estimate grazing intensity, we calculated percent use of herbaceous vegetation (*U*) as:

$$U\left(\%\right)=\left[\frac{I-O}{I}\right]*100$$

where *I* was the standing crop of herbaceous vegetation in the grazing exclosure and *O* was the standing crop of herbaceous vegetation in the paired grazed sampling area. We considered *U* to be an estimate of the intensity of grazing on herbaceous vegetation in the study sites; hereafter, we refer to *U* as grazing intensity. Herbaceous vegetation utilization was bound between 0 and 100% when there was greater standing crop of herbaceous vegetation in the grazing exclosure compared to the grazed paired area. However, in around 26% of the paired exclosure and grazed plots standing crop of herbaceous vegetation was greater in a grazed plot than in the grazing exclosure. Negative values for *U* could exceed 100%. Negative values for *U* are a consequence of inherent variation; zeroing or deleting negative consumption values results in overestimation of utilization (Bork & Werner, 1999; Hines et al., 2022). Instead of zeroing or deleting negative values, we scaled negative values so they were also bound between 0 and 100%. We did this by multiplying each negative value by 100 divided by the absolute value of the minimum negative use value obtained during our study (Hines et al., 2022).

To test our first and second hypotheses, we used negative binomial regression (Stoklosa et al., 2022) to examine the relationship between herbaceous plant species richness/0.25 m^2^ and the independent variables grazing intensity, percent sand, and the sum of precipitation during the 2 months (August and September) prior to vegetation sampling. Negative binomial regression analyses count data on the scale of measurement where transformations of count data perform poorly in regression models (O'Hara & Kotze, 2010). In addition, the estimated dispersion parameter of our models was significant (*p* < .001). Negative binomial regression accounts for effects of overdispersion (Oberle et al., 2009; SAS/ETS, 2017). We examined the relationship between covariates and the dependent variable herbaceous species richness/0.25 m^2^ using mixed models with random effects (PROC GLIMMIX, SAS 9.3; SAS Institute Inc., 2014). We used the Laplace approximation to the likelihood function and a random intercept with exclosure as the subject (Fitzmaurice et al., 2011). Covariates included in models were grazing intensity, grazing intensity^2^, percent sand (*S*), the sum of precipitation during the 2 months prior to vegetation sampling (*R*), grazing intensity×S, grazing intensity×R, grazing intensity^2^ × *S*, and grazing intensity^2^ ×R. We included squares of grazing intensity in models to test for quadratic relationships between grazing intensity and plant species richness; candidate models respected the principle of model hierarchy (Peixoto, 1990). We used backward selection starting with a model that included all covariates to choose the most competitive model in the candidate set. We used the Schwarz‐Bayesian information criterion to determine the best combination of covariates for predicting herbaceous species richness/0.25 m^2^ (Schwarz, 1978). The Bayesian information criterion often performs better than the Akaike information criterion or the Akaike information criterion corrected for small sample sizes when heterogeneity in the data set is large (Brewer et al., 2016). We did not consider a covariate to be influential when the 85% confidence interval overlapped 0 (Arnold, 2010). We standardized regression estimates using the STDCOEF option in SAS (SAS Institute Inc., 2014) with residual pseudolikelihood estimation. Standardization allowed us to compare the strength of the regression relationship among covariates that were not measured with the same units.

We estimated condition indices and variance inflation factors (VIF) for all models to assess multicollinearity among covariates (Belsley et al., 1980). We use multiple regression models to estimate condition indices and VIF with the covariates identified in the best model (SAS Institute Inc., 2014). In our models, the condition index with all independent variables included was 29.8. A condition index near 10 suggests weak dependencies; an index value >100 indicates regression estimates may have a numerical error (Belsley et al., 1980). Values for VIF were 1.020–1.023.

To test our third hypothesis that the effect of increasing ungulate grazing intensity on herbaceous plant species richness depends on patch productivity, we split our data into 2 bins based on herbaceous standing crop within grazing exclosures: <15.7 g/0.25 m^2^ and ≥15.7 g/0.25 m^2^. We conducted negative binomial regressions using PROC GLIMMIX (SAS Institute Inc., 2014) to examine the relationship between herbaceous plant species richness and influential covariates identified for the full data set. We then compared $\hat{\beta }$ of negative binomial regressions for each bin to test for unequal slopes using bins as class variables in negative binomial logistic regressions (Graybill, 1976; SAS Institute Inc., 2009).

## 3 RESULTS

### 3.1 Full data set

**FIGURE 2:**
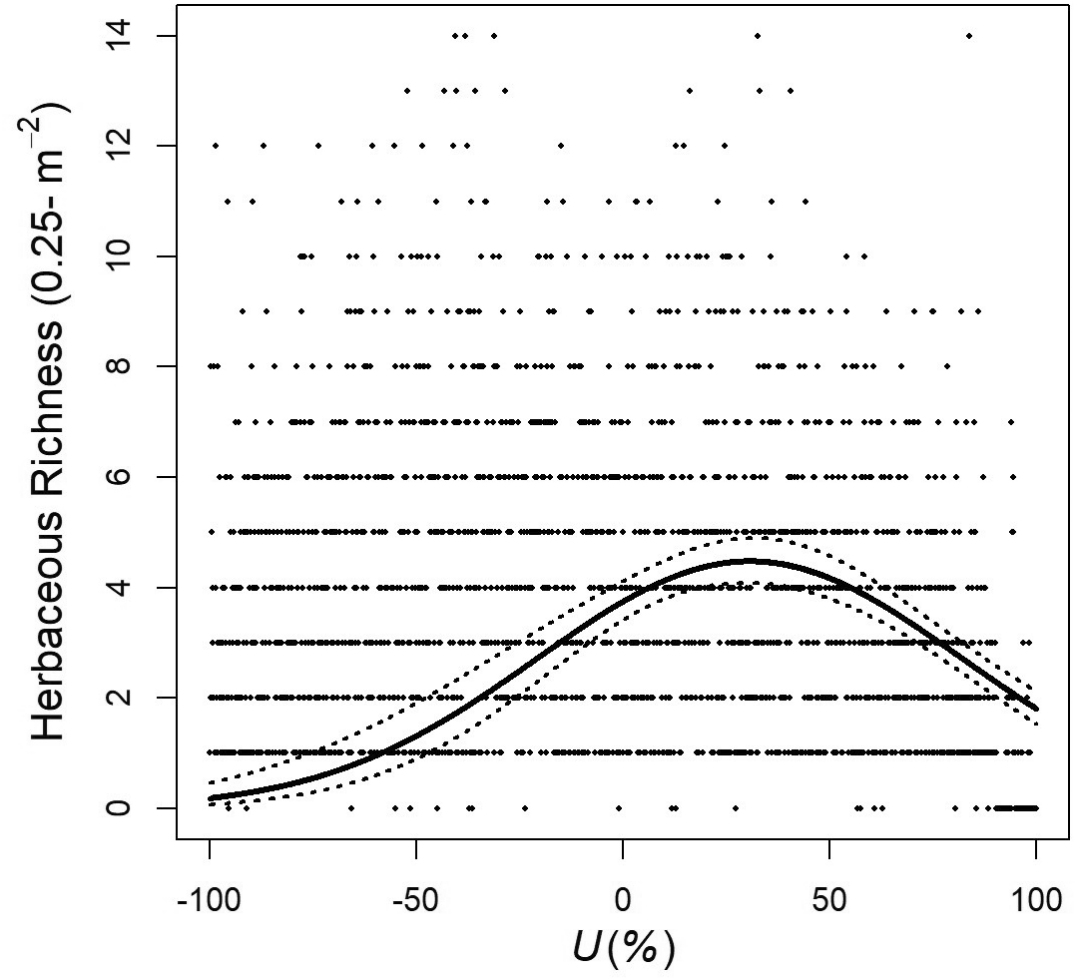
Relationship between mean herbaceous plant species richness/0.25 m^2^ and grazing intensity (*U*) across six study sites during 2012–2019 after adjusting for mean rain (165 mm) and percent sand (90%) to simplify presentation, South Texas, USA. The solid line represents values predicted by the model $\hat{Y}$ = −1.162 + 0.005 × rain +0.0266 × percent sand +0.012 × grazing intensity – 0.0002 × grazing intensity^2^. Dotted bands on either side of the line represent 95% confidence interval bands for predicted values. Black dots represent actual values.

The best model for predicting species richness/0.25 m^2^ included the covariates grazing intensity (standardized $\hat{\beta }$ = 19.0, 85% CL = 16.0–22.0, *p* < .001, *F*_1,1865 df = 82.2_), grazing intensity^2^ (standardized $\hat{\beta }$ = −28.4, 85% CL = −31.6 to −25.2, *p* < .001, *F* = 162.6), rain (standardized $\hat{\beta }$ = 2.4, 85% CL = 1.5–3.2, *p* < .001, *F* = 16.7), and percent sand (standardized $\hat{\beta }$ = 9.9, 85% CL = 8.3–11.4, *p* < .001, *F* = 82.1; Table 1). A model that included rain × use (standardized $\hat{\beta }$ = −0.2, 85% CL = −2.1 to 1.8, *p* = .882, *F* = 0.02) in addition to grazing intensity, grazing intensity^2^, rain, and percent sand was competitive but the 85% confidence interval of rain × use overlapped 0. Herbaceous plant species richness increased with increasing precipitation and percent sand holding other effects constant. However, grazing had a much stronger effect on species richness/0.25 m^2^ than precipitation or percent sand based on standardized $\hat{\beta }$ values. After accounting for the effect of rain and percent sand, herbaceous plant species richness/0.25 m^2^ exhibited a hump‐back response to increasing grazing intensity: estimated herbaceous plant species richness increased from 0 up to ~30% grazing intensity (where estimated richness was 4.5 ± 0.1 species/0.25 m^2^ and then declined with increasing grazing intensity (Figure 2)).

**TABLE 1:** Bayesian information criterion (BIC) for models predicting herbaceous species richness/0.25 m^2^, 2012–2019, South Texas, USA. A difference of 1–3 BIC values between competing models is weak evidence that the model with the smaller BIC is better, 2 and 6 is positive evidence, 6 and 10 is strong evidence, and >10 is very strong evidence (Raftery, 1995).

BIC	Variables in model
9419.47	Rain + percent sand + grazing intensity + grazing intensity^2^
9424.74	Rain + percent sand + grazing intensity + grazing intensity^2^ + rain × grazing intensity + rain × grazing intensity^2^
9425.15	Rain + percent sand + grazing intensity + grazing intensity^2^ + rain × grazing intensity
9430.21	Percent sand + grazing intensity + grazing intensity^2^
9430.37	Rain + percent sand + grazing intensity + grazing intensity^2^ + rain × grazing intensity + rain × grazing intensity^2^ + rain × percent sand
9430.71	Rain + percent sand + grazing intensity + grazing intensity^2^ + rain × grazing intensity + rain × percent sand
9432.6	Rain + percent sand + grazing intensity + grazing intensity^2^ + rain × grazing intensity + rain × percent sand + rain × percent sand^2^
9435.15	Rain + percent sand + grazing intensity + grazing intensity^2^ + rain × grazing intensity + rain × grazing intensity^2^ + rain × percent sand + rain × percent sand^2^
9491.08	Rain + grazing intensity + grazing intensity^2^
9496.34	Rain + grazing intensity + grazing intensity^2^ + rain × grazing intensity + rain × grazing intensity^2^
9582.83	Rain + percent sand + grazing intensity
9586.84	Rain + percent sand + grazing intensity + rain × grazing intensity

### 3.2 Split data set

**FIGURE 3:**
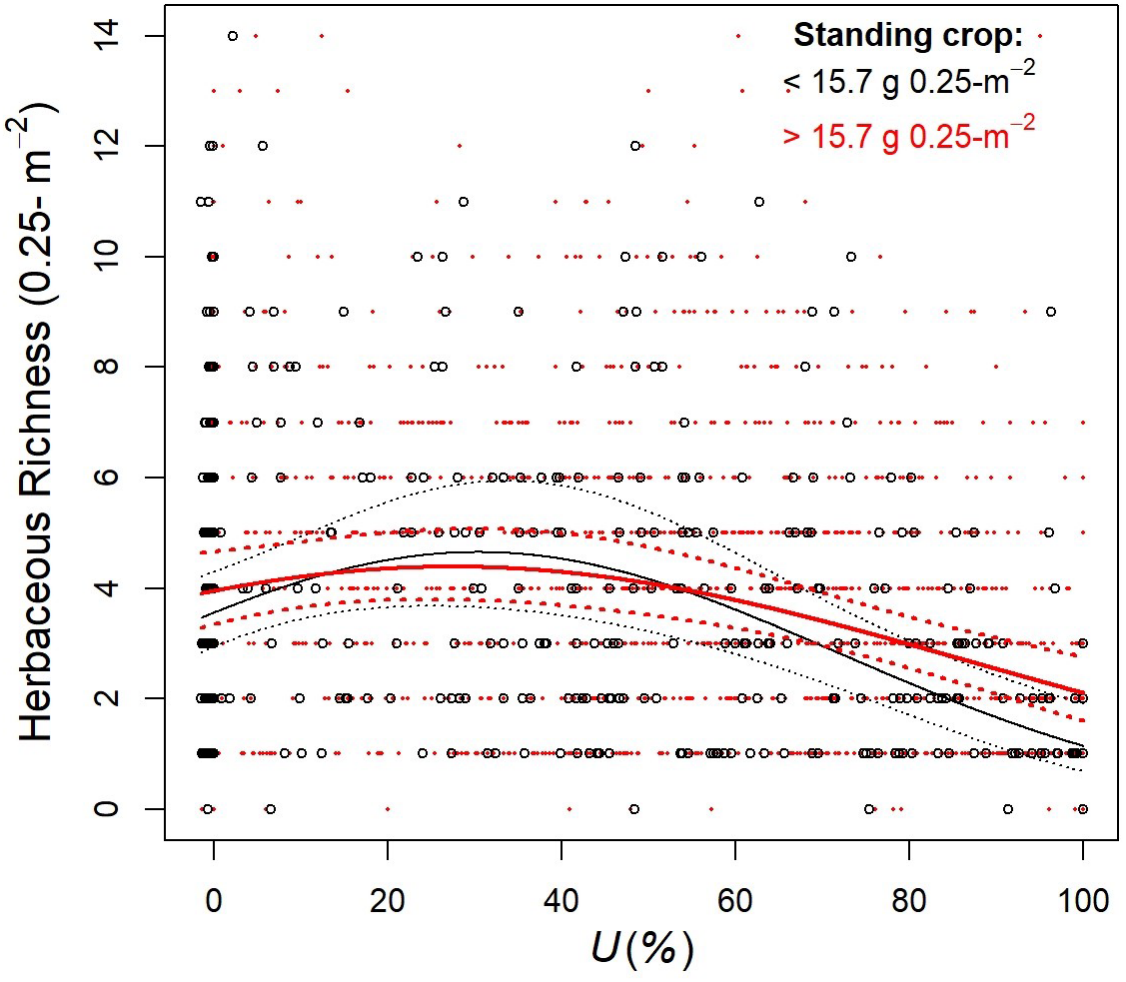
Relationship between mean herbaceous plant species richness/0.25 m^2^ (±95% confidence intervals) and grazing intensity (*U*) across six study sites during 2012–2019 with standing crop <15.7 g/0.25 m^2^ (black line) or ≥15.7 g/0.25 m^2^ (red line) in exclosures after adjusting for mean rain (165 mm) and percent sand (90%) to simplify presentation, South Texas, USA. The solid lines represent values predicted by the model $\hat{Y}$ = −0.035 + 0.001 × rain +0.013 × percent sand +0.018 × grazing intensity – 0.0003 × grazing intensity^2^ for standing crop <15.7 g/0.25 m^2^ and $\hat{Y}$ = − 1.506 + 0.002 × rain +0.032 × percent sand +0.008 × grazing intensity – 0.0001 × grazing intensity^2^ for standing crop ≥15.7 g/0.25 m^2^. Dotted bands on either side of the lines represent 95% confidence interval bands for predicted values. Black and red dots represent actual values.

Out of 2400 grazing exclosures that we sampled during 2012–2019 on 6 sites, 35% had standing crops <15.7 g/0.25 m^2^ (Table 2). Percentage of protected plots with standing crops <15.7 g/m^2^ was highly heterogeneous in space and time, ranging from 4 to 100 depending on site and year. Averaged across sites and years of study, standing crop of herbaceous vegetation was 75% greater in grazed plots than in protected plots in the least productive patches; the opposite occurred in the more productive patches (Table 3). Here, patch refers to a paired protected and unprotected plot. Regression models differed (*p* < .001, *F*_5,1860 df = 12.8_) between the least productive patches and more productive patches. Additionally, intercepts (*p* = .001, *F*_1,1860 df = 10.2_) differed and regression planes were not parallel (*p* < .001, *F* = _11,864 df = 11.6_). The slope of the regression line for the least productive patches (<15.7 g/0.25 m^2^) differed (*p* < .001, *F*_1,1864 df = 18.9_) from slope of the regression line of the more productive patches (>15.7 g/0.25 m^2^). After accounting for the effect of rain and percent sand, linear and quadratic responses of herbaceous plant species richness to increasing grazing intensity were greater for the least productive patches than for the more productive patches (Table 4, Figure 3). Herbaceous species richness increased with increasing grazing intensity, reaching a maximum estimated 4.70 ± 0.23 species/0.25 m^2^ at ~30% grazing intensity in less productive (<15.7 g/0.25 m^2^) patches and 4.43 ± 0.13 species/0.25 m^2^ at ~27% grazing intensity in more productive (>15.7 g/0.25 m^2^) patches. At higher grazing intensity, herbaceous species richness declined with increasing grazing intensity more steeply in less productive patches than it did in more productive patches.

**TABLE 2:** Percentage of plots on East Foundation ranches with standing crop <15.7 g/0.25 m^2^ within exclosures during 2012–2019, South Texas, USA.

Site	g/0.25 m^2^	2012	2013	2014	2015	2016	2017	2018	2019
		% of plots							
Buena Vista	<15.7	88	34	8	100	20	12	26	20
East El Sauz	<15.7	36	18	12	44	16	34	14	42
San Antonio Viejo (1)	<15.7	42	64	4	6	16	70	40	60
San Antonio Viejo (2)	<15.7	64	64	44	10	10	74	18	66
San Antonio Viejo (3)	<15.7	38	48	44	42	32	40	40	34
Santa Rosa	<15.7	70	16	6	18	18	72	26	56

**TABLE 3:** Mean number of herbaceous plant species/m^2^ and 95% confidence intervals and mean standing crop (g/0.25 m^2^) and 95% confidence intervals in exclosures and grazed plots in patches where standing crop within exclosures was <15.7 g/0.25 m^2^ or ≥15.7 g/0.25 m^2^ during 2012–2019 averaged across years, South Texas, USA.

Parameter	*n*	Exclosure		Grazed	
		Mean	95% CI	Mean	95% CI
	<15.7 g/0.25 m^2^				
Number of species/0.25 m^2^	840	3.2	3.0–3.4	2.8	2.6–3.0
Standing crop (g/0.25 m^2^)	840	5.9	5.5–6.2	10.3	9.1–11.5
	≥15.7 g/0.25 m^2^				
Number of species/0.25 m^2^	1560	4.7	4.5–4.8	4.1	3.9–4.2
Standing crop (g/0.25 m^2^)	1560	48.6	46.7–50.6	29.1	27.7–30.6

**TABLE 4:** Standardized slope estimates ($\hat{\beta }$) and 95% confidence intervals for grazing intensity (*U*, %), grazing intensity^2^, rain, and percent sand where standing crop within exclosures was <15.7 g/0.25 m^2^ or ≥ 15.7 g/0.25 m^2^ averaged across ranches and years, South Texas, USA, (2012–2019). Estimates are based on analysis of each subset of standing crop.

Standing crop	*U*		*U* ^2^		Rain		Sand (%)	
g/0.25 m^2^	$\hat{\beta }$	95% CI	$\hat{\beta }$	95% CI	$\hat{\beta }$	95% CI	$\hat{\beta }$	95% CI
<15.7	17.3	12.3–22.3	−25.0	−31.0 to 22.2	2.2	0.9–3.5	2.7	1.1–4.3
≥15.7	10.1	6.2–14.1	−17.4	−21.5 to −13.2	0.9	−0.2–2.0	9.5	7.5–11.6

## 4 DISCUSSION

Our results do not agree with the conclusion of Gao and Carmel (2020b) that the MSL model is a better predictor of the effect of grazing on plant species richness than the IDH model on rangelands. Instead, our results corresponded to the pattern predicted by the IDH model in that small‐scale herbaceous plant species richness exhibited a quadratic response to increasing ungulate grazing intensity. Additionally, our results differed from conclusions of 2 recent meta‐analyses of grazing effects on plant species richness. Contrary to our findings, Gao and Carmel (2020b) concluded that predictions of the IDH model were not supported in arid and semiarid areas. Results of the meta‐analysis by Herrero‐Jáuregui and Oesterheld (2018) differed from those of Gao and Carmel (2020b) and from our findings in that stocking rate had little effect on species richness even on productive sites. Both Gao and Carmel (2020b) and Herrero‐Jáuregui and Oesterheld (2018) concluded that grazing had negative effects on species richness in dry areas. Our results from an environment that is dry in comparison to areas where increases in species richness at intermediate intensities of disturbance were documented show that the IDH model is possibly more robust across climatic regions than suggested in other studies (Baaker et al., 2006; Sasaki et al., 2009). However, we did not examine mechanisms such as competition among plants that are predicted by the IDH model. Consequently, we can only state that the pattern of species richness responses to grazing corresponded to the IDH model but whether or not the pattern is the result of mechanisms predicted by the model cannot be determined from the data we collected.

There are several possible reasons our results differed from other published studies. Experimental designs vary greatly among studies, ranging from large fenced and unfenced plots to grazing gradients based on distance to water (Baaker et al., 2006; Gao & Carmel, 2020b; Herrero‐Jáuregui & Oesterheld, 2018; Sasaki et al., 2009). Spatial and temporal scale of sampling, grazing history, and other aspects of methodology also vary greatly among studies. Spatial replication conducted over multiple years as was the case in our study is uncommon in the literature (Herrero‐Jáuregui & Oesterheld, 2018). In addition, grazing intensity is often categorized as “light,” “moderate,” or “heavy” based on local context rather than direct, quantitative estimates such as that used in our study. For example, Herrero‐Jáuregui and Oesterheld (2018) converted stocking rates into grazing intensity classes (low, moderate, and high) rather than using actual estimates of grazing intensity as we used. Species of grazers and combinations of grazers vary widely across studies and type of herbivore may influence results (Gao & Carmel, 2020b; Olff & Ritchie, 1998). Large grazing herbivores, for example, have more consistent effects on plant species diversity than small and intermediate‐sized herbivores (Olff & Ritchie, 1998).

Estimates of plant species richness are sensitive to spatial scale of vegetation sampling, which may influence variation in results among studies. For example, plant species richness in 1‐m^2^ plots was greater in grazed sites than in exclosures in Rocky Mountain grasslands, but there was no difference in plant species richness between grazed and protected plots when 1000‐m^2^ plots were sampled (Stohlgren et al., 1999). Our results therefore only apply to our scale of measurement (0.25 m^2^) and we acknowledge that our results could have differed if we had used larger scales (e.g., 10, 100, or 1000 m^2^).

We rejected our second hypothesis that precipitation and percent sand interact with grazing intensity. In our study, precipitation had a weaker influence on herbaceous plant species richness than percent sand or grazing intensity. Reported relationships between plant species richness and annual precipitation vary in the literature. For example, in the annual grasslands of California, USA, variation in species richness corresponded to changes in mean annual precipitation (Fernandez‐Going et al., 2012). In contrast, Cleland et al. (2013) reported positive relationships between mean annual precipitation and species richness across sites, but few relationships within sites, in 10 grasslands ranging from California to Michigan in the United States. Similar to our findings, Herrero‐Jáuregui & Oesterheld, (2018) concluded that the response of plant species richness to stocking rate was not related to mean precipitation.

We expected a stronger relationship between precipitation and herbaceous plant species richness because vegetation dynamics may be more closely linked to variation in precipitation than to herbivory in environments with low and highly variable precipitation (Briske et al., 2003; Derry & Boone, 2010; DeYoung et al., 2019; Ellis & Swift, 1988). Several authors have suggested a threshold of ~33% coefficient of variation in annual precipitation above which the linkage between vegetation dynamics and herbivory decouples (Derry & Boone, 2010; Ellis & Swift, 1988; Vetter, 2005). Ecosystems with >33% coefficient of variation in annual precipitation are likely to exhibit nonequilibrium or disequilibrium vegetation dynamics rather than equilibrium dynamics or directional change in vegetation in response to disturbance. The intermediate disturbance hypothesis assumes directional succession, that is, equilibrium vegetation dynamics. Coefficient of annual variation in precipitation in our study area exceeded 33% on all of our study sites except the Santa Rosa and El Sauz ranches. If vegetation and herbivores are decoupled in such environments, we would expect the assumptions of the IDH model to not apply. Yet, even though most of the study sites were near or in excess of the 33% coefficient of variation in annual precipitation threshold, herbaceous plant species richness appeared to be linked to herbivory. Our results followed the pattern predicted by the IDH model for response of small‐scale herbaceous species richness to grazing even in an ecosystem where nonequilibrium vegetation dynamics might be expected.

Herbaceous plant species richness was greater in sandier soils similar to the findings of other researchers (Pennington et al., 2017; Sanaei et al., 2019); however, the relationship between species richness and grazing intensity did not vary with percent sand as we predicted. A possible reason for the positive relationship between herbaceous plant species richness and percent sand is that sandier soils often have greater soil water availability in semiarid environments (Noy‐Meir, 1973; Sala et al., 1988). Soils higher in clay experience greater evaporation than sandy soils resulting in more rapid soil moisture depletion.

Our results did not support our prediction that small‐scale herbaceous plant species richness is more strongly related to ungulate grazing intensity in productive patches than in sparse patches of vegetation. Other researchers have reported that grazing decreased species richness in areas with low productivity, but increased species richness in areas of high productivity (Baaker et al., 2006; Lezama et al., 2014). Standing crop of herbaceous vegetation in our study sites was within the range of biomass values considered to be characteristic of areas with low productivity by both Baaker et al. (2006) and Lezama et al. (2014).

Reasons for greater standing crop in the grazed plots than in the protected plots in the least productive patches are unclear. Trampling by sheep increased seedling emergence in dry open sand ecosystems (Eichberg & Donath, 2018). In our study, trampling by large ungulates possibly had a similar effect in relatively barren patches but not in patches with greater herbaceous standing crop. Another possibility is compensatory growth of plants in sparse vegetation patches in response to herbivory (Gruntman & Novoplansky, 2011). Gruntman and Novoplansky (2011) found that vegetation overcompensated in the least productive patches and undercompensated in response to grazing in the most productive patches.

Our results agreed with conclusions of Herrero‐Jáuregui and Oesterheld (2018) that herbaceous species richness declines with increasing grazing intensity after “moderate” grazing intensity is reached. In addition, the steeper decline in species richness as grazing intensity increased beyond “moderate” levels in our study was similar to results at larger spatial scales reported by other researchers (Herrero‐Jáuregui & Oesterheld, 2018; Milchunas et al., 1988).

## AUTHOR CONTRIBUTIONS

**Timothy E. Fulbright:** Conceptualization (equal); data curation (equal); formal analysis (equal); funding acquisition (equal); investigation (equal); methodology (equal); project administration (equal); supervision (equal); visualization (equal); writing – original draft (equal). **J. Alfonso Ortega‐Santos:** Conceptualization (equal); data curation (equal); funding acquisition (equal); investigation (equal); methodology (equal); project administration (equal); resources (equal); supervision (equal); writing – review and editing (equal). **Stacy L. Hines:** Data curation (equal); formal analysis (equal); investigation (equal); methodology (equal); project administration (equal); supervision (equal); writing – review and editing (equal). **Dillan J. Drabek:** Data curation (equal); investigation (equal); writing – review and editing (equal). **Ramon Saenz:** Data curation (equal); investigation (equal); supervision (equal); writing – review and editing (equal). **Tyler A. Campbell:** Funding acquisition (equal); resources (equal); writing – review and editing (equal). **David G. Hewitt:** Project administration (equal); supervision (equal); writing – review and editing (equal). **David B. Wester:** Data curation (equal); formal analysis (equal); writing – review and editing (equal).

## CONFLICT OF INTEREST STATEMENT

None declared.

## Data Availability

The data that support the findings of this study are openly available in Dryad at https://doi.org/10.5061/dryad.2bvq83bww.
